# Experimental Implementation and Performance Evaluation of an IoT Access Gateway for the Modbus Extension

**DOI:** 10.3390/s21010246

**Published:** 2021-01-01

**Authors:** Vasile Gheorghiță Găitan, Ionel Zagan

**Affiliations:** 1 Faculty of Electrical Engineering and Computer Science, Stefan cel Mare University, 720229 Suceava, Romania; 2 Integrated Center for Research, Development and Innovation in Advanced Materials, Nanotechnologies and Distributed Systems for Fabrication and Control (MANSiD), Stefan cel Mare University, 720229 Suceava, Romania

**Keywords:** local industrial networks, Modbus and Modbus extension, acquisition cycle, IoT gateway

## Abstract

This paper presents the relevant aspects regarding the experimental implementation and performance evaluation of an Internet of things (IoT) gateway for the Modbus extension. The proposed Modbus extension specifications are extended by defining the new optimized message format, and the structure of the acquisition cycle for obtaining a deterministic temporal behavior and solutions are presented for the description of devices at the MODBUS protocol level. Three different implementations are presented, and the Modbus extension’s performance is validated regarding the efficiency in the use of the acquisition cycle time. The software and hardware processing time and the importance and effect of the various components are analyzed and evaluated. They all support the implementation of an Internet of things gateway for Modbus extension. This paper introduces solutions for the structure of the acquisition cycle to include other valuable extensions, discusses the performance of a real implementation in the form of a gateway, adds new features to the Modbus extension specification, and strengthens some of the existing ones. In accordance with the novelty and contribution of this paper to the field of local industrial networks, the results obtained in the analysis, testing, and validation of the Modbus extension protocol refer to the extending of the Modbus functions for industrial process monitoring and control management.

## 1. Introduction

Few developments, such as the introduction of networks as a support for communication of information between devices (local industrial network; LINW) have changed the face of automation profoundly. They provide physical and logical support to create data acquisition channels that depart from sensors and transducers and must end with integration into Internet of things (IoT), especially industrial IoT (IIoT). LINW is a comprehensive term that includes networks on a sensor/actuator level (sensor bus, fieldbus, or field area network (FAN)) that connect smart sensors and execution elements with 4–16 digital inputs/outputs (AS-I, CANOpen, Modbus RTU, Lonworks), those on a device level (device bus) that connect groups of 32–256 digital inputs/outputs and small automation units (Profibus, Interbus-S, CANOpen, Modbus, etc.), and those on a control level, where the best networks can connect data concentrators, automation units, controllers for CNC machines, PCs, HMI, and PLCs, with many inputs/outputs (industrial Ethernet, EtherCAT, EPL, Sercos III, CC-Link, Profinet, ControlNet, WorldFIP, and Modbus TCP/IP).

There are also LINWs (fieldbuses) oriented for specific applications or domains. The following examples may be given: the automotive industry, “smart” homes, centralized management of a building, movement control, networks implementing measurement and testing techniques, and the military or the air force, where stringent reliability restrictions are imposed [1]. In recent years, LINW concerns have consistently remained at a high level. As a result, a multitude of specifications and communication protocols have emerged, either free or private. Due to their accumulation, the efforts of specialists in the field are currently aimed toward standardization and reducing the number of standards. From the outset, it is worth pointing out that the evolution of industrial Ethernet will have a major influence in the further development of LINWs. However, LINWs [2] are much better optimized for specific automation tasks than those based on Ethernet, and implementation is cheaper. In this context, not all protocols have complete specifications, such as the Modbus protocol, M-bus, and the ASCII protocols. As a result, time-coherence issues, MAC (Medium Access Control) schemas, and descriptions of networked devices may occur. The Modbus RTU is such a protocol. The Modbus RTU is an LINW protocol that can be used at the sensor/actuator level and device level (the Modbus TCP/IP specification can also be used for the control level). The Modbus protocol was created in 1978 by Modicon Inc. as an easy way to communicate control data between controllers and sensors using an RS232 port. The protocol was then widely adopted, rapidly achieving de facto standard status in the field of industrial automation. Today, the Modbus protocol is a unique protocol, one of the most appreciated and used among automation devices. Schneider Electric transferred the Modbus and Modbus/TCP (Modbus on TCP/IP) specifications to Modbus.org. For client/server communication between devices connected to different types of buses or networks, Modbus uses an application layer, a protocol placed on Level 7 of the OSI stack. Modbus is currently transported using any of the following: RS232, RS422, RS485, TCP/IP, Modbus Plus, which is a chip transmission network, and many other stacks on a wide variety of media (e.g., optical fiber and GSM). Modbus’ popularity stems from a commitment to simplicity, while acknowledging that industrial automation applications are very diverse and that there are advantages in delegating diversity manipulation to applications.

In this paper, we present our applied research on the Modbus protocol on issues of temporal coherence with the definition of an acquisition cycle (AC), the optimization of the use of bandwidth, and the definition of a device description language according to the results presented in [3]. Here, an original extension called ModbusE (Modbus extension) is proposed. In this paper, we use the abbreviation MBE. As the main contribution, we implemented the structure of an acquisition cycle for incompletely defined networks in order to add a time stamp and to achieve the broad temporal coherence. This paper is a continuation of a previous work [3] regarding MBE and acquisition cycle for incompletely defined protocols. The solutions proposed in this paper are simple and low-cost, because they allow the integration of existing acquisition modules without changes in terms of hardware or software. The following Modbus aspects contributed to the practical results presented in this paper: CRC calculation, measuring time intervals 3.5 and/or 1.5 between two consecutive characters, an algorithm that determines the time period of the slots, defining public commands (100–102) to perform slot map definitions, and reading operations, as well as the physical setting functions of Modbus slots and addresses. All these characteristics are addressed below by explicitly presenting the contribution of the authors to the development and definitions of the basic aspects of the MBE protocol. 

The first section of the paper contains a brief introduction, highlighting the contributions of the authors. Section 2 describes similar papers in the field of LINWs based on the MODBUS protocol. Section 3 is dedicated to the Modbus extension on which the particular results of this protocol implementation are based, and Section 4 describes the validation of the proposed MBE concept. Section 5 focuses on discussions regarding the practical evaluation of the MBE and the integration of BSG (Base Station Gateway) in IoT. The paper ends with the final conclusions in Section 6.

## 2. Related Works

The most recent Modbus update dates back to 2012, and a new security specification appeared in Modbus TCP/IP in 2018. Scientific papers related to Modbus refer in particular to Modbus TCP/IP addressing issues related to the following, among others: use as a support for communication in distributed applications [4] (mostly SCADA [2]), the implementation of access gateways (for remote connection using a TCP/IP protocol stack [5] and wireless connection implementation [6]), security and authentication [7], the detection, simulation, and modeling of unwanted attacks, anomalies, and intrusions [8], vulnerabilities [9,10], software for integration into the OPC UA industrial middleware [11], performance analysis in the context of the complexity of the TCP/IP stack [12], and network traffic simulation [13].

The IoT access gateway is equipped with a MODBUS TCP/IP server. Server UA OPC deployments have an interface commonly called Data Provider that makes a wrapper between the server and the various drivers for LINW including the well-known MODBUS TCP/IP client driver. Through the OPC UA server, the gateway can be accessed from IoT applications. The recent PubSub (publisher/subscriber) specification for OPC UA has been expanded so that, in addition to the classic client–server protocols (TCP/IP and HTTP/SOAP), publisher/subscriber communications can also be made. Specifically, PubSub allows the use of OPC UA directly on the Internet (wide area networks) by using popular IoT-specific data protocols such as MQTT and AMQP, while retaining the end-to-end OPC UA security key and the advantages of data modeling standardization. Similarly, PubSub also allows the use of user data protocol (UDP) to establish low-delay connections, which tolerate losses on LAN networks. Thus, the OPC UA combines both communication paradigms. The question of choosing among “OPC UA, AMQP, or MQTT” does not count, says the OPC Foundation, because OPC UA can also deliver this. However, a device with limited resources with “MQTT only” should provide its data in the format of the information model “OPC UA via MQTT”.

A central challenge of Industry 4.0 and IIoT is the standardization and security of data and information exchanged among devices, machines, and services, even in different industries. In April 2015, the Industry 4.0 Reference Architecture Model (RAMI 4.0) already listed IEC 62,541 OPC United Architecture (OPC UA) as the only recommended solution for implementing the communication layer. The basic requirement for using OPC UA for industrial communications 4.0 is an Internet Protocol (IP)-based network. Anyone wishing to advertise with the label “Industry 4.0-capable” must also be capable of OPC UA (integrated or through a gateway). The ownership of the information modeling of the UA OPC is explicitly highlighted.

In this paper, the focus is Modbus RTU. The scientific work related to Modbus RTU refers primarily to the use of this protocol as a serial communication medium for different fields of application, not only for monitoring and directing industrial processes [14] but also for the automation of houses, buildings [15], electricity transmission [16], etc.

Another research and development direction for Modbus RTU refers to methods of detection and correction of errors. In [17], error detection was performed using a specialized repeater device in the receiver and transmitter. This method complies with the parameters of the Modbus RTU protocol; thus, the bus extension with the retransmission devices can also be achieved with normal Modbus RTU devices. Reed–Solomon codes were chosen from different methods of the detection and correction of errors, and these methods are systematic and can correct errors at the level of isolated bits, as well as errors given by pulses. Another concern in this area of research is the productivity of the software, its easy maintenance, and its reuse of the code to obtain many of the desired properties of industrially integrated network software.

In [18], the authors described a case study of the Protege language in an industrial setting. The authors implemented the Modbus protocol on TCP/IP and serial lines and tested it using an industrial gateway. The implementation described in [18] demonstrates the advantages of Protege, and the main technical contribution made by the authors was the exemplification of the decomposition of the functionality of a typical industrial protocol, which improved the modularity and reuse of the code. It was demonstrated that the use of Protege greatly facilitates the implementation of the protocol stack, increases the sharing and reuse of the code, and makes maintenance much easier. Reference [19] introduced and presented the model of the Modbus slave protocol based on Modbus/RS-485 and its implementation. This work focused on designing a new software architecture, with the execution of the program being coordinated by a real-time operating system (RTOS). ARM (Advanced RISC Machines) hardware was designed and implemented to verify the operation and performance of the software. By designing a new software architecture and integrated stacks, the time period corresponding to the development was reduced, and the software was easy to port, maintain, and reuse.

Adapting network levels and implementing gateways between protocols is another area of research in Modbus RTU. In [20], the authors proposed a Modbus adaptation level for the controller area network, called MODBUS CAN, which was formally verified and evaluated experimentally. The results presented by the authors showed that, in a typical low-cost built-in system, MODBUS CAN performance compares favorably with regard to a Modbus TCP implementation, based on an Ethernet network with a 100 Mbps transfer rate, performed on the same system and using the same set of protocols. However, the comparison does not refer to the same class of protocols. The paper describes the design and validation of MODBUS CAN, the main purpose of which is to fragment and reassemble MODBUS protocol data units (PDUs) (up to 253 bytes in length) and, thus, fit them into CAN frames (which hold up to 8 bytes of payload). Compared to Modbus RTU, MODBUS CAN delivers considerably higher performance, while retaining similar implementation costs and simplified wiring based on bus topology. The claim is not supported and, in [3], is somewhat countered.

Reference [21] proposed an architectural improvement of the Modbus RTU protocol for the integration of equipment into industrial automation networks, using hybrid communications with wired Modbus RTU and wireless IEEE 802.15.4. The proposed hybrid communications protocol increases the control and topological limits imposed by Modbus RTU, allowing for a wired/wireless tree-bus topology and master multiplexing. On the basis of the tests, the proposed architecture showed a low rate of communication error, indicating that the developed solution can meet the robust requirements of industry communications networks.

In [22], the authors presented a response and scheduling time analysis tool for Modbus communications on RS485 networks. The response times of a message set were collected by a specialized Modbus device and then sent to the software performing the analysis. To evaluate the developed tool, a Modbus application was implemented on the RS485 network in a laboratory environment. The paper discussed the development of a tool to assess real-time requirements in Modbus RS485 networks. The authors’ proposal involved evaluating response times in two approaches. In the first approach, the Master Device of the Modbus network was replaced with a specialized device capable of assessing response times for a set of known messages. In the second approach, the specialized device acted as a passive device that was connected to the network and collected information about messages exchanged between devices and their response times. Using statistics, charts, and lists, information about response time, periods, and message content was displayed, helping the user with different ratings. Using the proposed tool, three case studies were carried out to verify the communication characteristics and query set, thus indicating the variation in response times according to device characteristics. However, in order to improve network characteristics for particular applications, issues involving time-outs and communication errors should also be taken into account. Due to the popularity of the Modbus protocol, various Modbus extensions have been proposed, and, for the most part, they retain compatibility with the specifications mentioned in Section 1. Some of these extensions are analyzed below.

In ref. [23], the authors first proposed the extension of the address space using a reserved address and then extended the address space from 8 to 16 bits. Only stations aware of this extension can understand this way of addressing those that are immune to these messages. The paper also proposed a multi-master architecture with the choice of the next master. To do this, a master choice protocol is periodically launched. The second function of this protocol is to make all master devices aware of the presence of others. This way, it can send them additional information about the current activity.

In ref. [3], an AC was presented in order to achieve an extended temporal coherence, which can also be customized for Modbus. An original protocol extension called ModbusE, based on multi-microprocessor (MM) working mode, was proposed that brought it close to the performance of the CANOpen protocol. In the ModbusE protocol, only the slot number, data, and cyclic redundancy check (CRC) are transmitted during a slot, thus increasing the bandwidth of the communication channel. The meaning of the data is defined by default in device configuration, or by classic Modbus commands when initializing the AC. A similar idea can be found in [24], but for CAN FD; in that paper, the authors proposed a solution for an extension of the CAN protocol (CAN) with extensible in-frame reply (XR) that allows higher levels of the protocol to define new dedicated user services for, e.g., network management, application-specific functions, and a data transfer with increased efficiency. At the application level, the management of variables and device parameters can be a challenge. In ref. [25], research focused on a universal method for describing protocols, and that method aimed to encapsulate packages, where various protocol messages can be encapsulated and analyzed by an interpreter in a unified way. To ensure communication efficiency and quality of service (QoS) for different types of messages, the encapsulation of packages using the protocol description was optimized and scheduled before transmission by the interpreter. A closer approach to Modbus was presented in ref. [26]. There are also implementation approaches in field-programmable gate arrays (FPGA) [27] or systems on chip (SoC) [28].

## 3. Modbus Extension—MBE

MBE was succinctly defined in ref. [3] specifying the structure of the message, the structure of the AC, BSG (base station gateway) as the Master device (we still call it Client) that manages the AC and provides a gateway to other protocols such as Modbus TCP-IP, PDO (process data object), and SDO (service data object), the use of new generations of microcontrollers (µCs) that can support speeds above 12 Mb/s (e.g., 27 Mb/s for STM32F756), MM work for UART (in this case only with MBE devices), interrupts, direct memory access (DMA) transfer, and RS485-specific facilities for changing the communication direction.

### 3.1. MBE Message Structure

**Figure 1:**
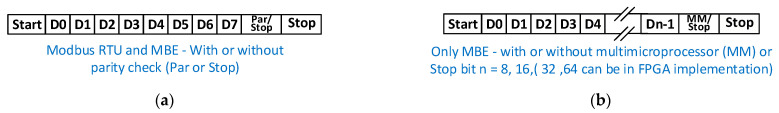
(**a**) Modbus and RTU and MBE frame with one parity bit and one stop bit only or with two stop bits; (**b**) MBE frame with MM and a stop bit or with two stop bits with 8, 16, 32, and 64 bits (data sizes are greater than 16 for FPGA implementation only).

There are three types of transactions in MBE: *Send Data with Acknowledgment* (SDA), which sends data with recognition (request and response), *Send Data with No Acknowledgment* (SDN), which sends data without recognition (request), and *Send and Request Data* (SRD), which sends and requests data (request–response). Figure 1a shows the frames sent on serial lines for both Modbus RTU and MBE. They can contain one bit of parity and one bit of stop, or only two bits of parity. Figure 1b shows specific MBE frames that have a bit for MM communication control and a stop bit, or only one or two stop bits. The data size is 8 bits in the µC (due to DMA transfer for characters exceeding 8 bits, 16 bits of data are rarely transmitted), and there can also be data sizes of 16, 32, and 64 bits for FPGA implementations.

**Figure 2:**
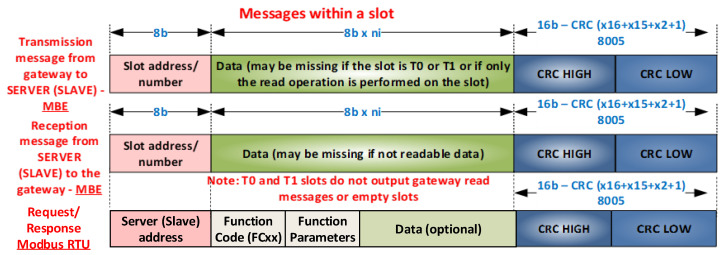
MBE; Modbus RTU messages.

Figure 2 shows the message structure for MBE. The first message is a request, the second is used for a response, and the structure of the Modbus RTU request/response message is illustrated by the third message. It is easy to see that MBE messages do not have the function code fields and function parameters in either the request message or the response message, and the required bandwidth is clearly lower than in the case of Modbus RTU; in other words, for the same useful amount of data, the MBE message is shorter than the Modbus RTU message (data structure and length information is either configured offline with simple Modbus configuration tools or carried out online by BSG). An MBE message easily defines a Modbus RTU message if the slot address becomes a server (slave) address and if the first part of the data range becomes the function code with function parameters. Additional MBE addresses larger than 0 × 80 are treated by default as Modbus RTU addresses.

As a result, an 8 bits Modbus RTU/MBE frame for 8 bits data has 11 bits (11b). A Modbus RTU message has a maximum of 256 bytes.

Modbus RTU (MMRTU) message = address (1×control frame (*cf*)) + function code (*fc*) (1 × control frame) + function parameters (*fp*) (fp × control frame) + data (*n* × data frame (*df*)) + CRC (2 × control frame) = >

(1) MMRTU_frame=(4+fp)×cf+n×d

(2) (cf=df=11 bits)×MMRTU_bits=(4+fp+n)×11 bits

A MBE message has a maximum of 256 bytes.

MBE (MMBE) message = address (1 × control frame (*cf*)) + data (*n* × data frame (*df*)) + CRC (2 × control frame) = >

(3) MBE_frame=3×cf+n×d

(4) (cf=df=11 bits)×MMBE_bits=(3+n)×11 bits

For the same amount of payload data,

(5) MMRTU_frame−MBE_frame=(4+fp−3)×cf+(n−n)×df=1+fp,  in bits: 11+fp×11 bits

### 3.2. Acquisition Cycle Architecture

**Figure 3:**
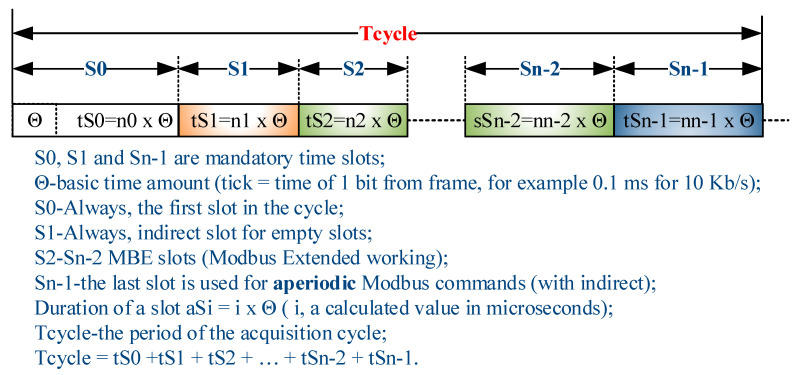
Acquisition cycle structure.

The structure of the AC is presented in Figure 3 and is based on the proposal presented in ref. [3], where S0 is SYNC and Sn-1 is SEND.

For the AC of gateMBEx, the following requirements are defined:

- The AC can have *n* slots, where *n* ϵ 3–46 (one less than the maximum number of Modbus addresses, 247, with the latter number being retained for local gateway commands), each slot supporting *n*_0_, *n*_1_, ..., *n*_*n*−1_ characters issued, except Slots 0, Slot 1, and those redirected to Slot 1.
- The minimum number of slots in an AC is 3.
- A slot is made up of *n_i_* × 11 × θ ticks (it is preferable that the tick does not generate interrupts but is the clock in the timer).
- Each slot has a data structure attached with status, control, and data information.
- A ModbusE (ModbusExtension) station, by configuration, can subscribe to multiple broadcast/reception slots.

There are three slots with special functions:

- Slot 0 (*n*_0_) is the slot that marks the beginning of the cycle and can be used, for example, to broadcast the “start scanning” inputs or update outputs.
- Slot 1 (*n*_1_) is used for indirect empty slots (either they have not been defined or the slaves working with these slots are stopped or defective). If indirectness is not carried out, sending the command could mislead those stations that subscribed to these slots. Slot 1 has no slave device connected; thus, it cannot be subscribed. When used for indirection, only the slot address and the CRC are sent, but its duration inherits the time period of the indirection. There is a byte that usually has a value of 0, but other values may have different meanings. For now, a value other than 0 in the range 1–127, but not greater than the number for Sn-1, can indicate a slot where there is a possible gateway client that can take over the gateway function and, thus, AC management.
- The last slot (*n*_*n*−1_) is used for aperiodic commands and is usually indirectly with Slot 1, if there are no aperiodic commands, or with a slot with a number greater than the last slot in the cycle containing the indirect command. This command must have a number of bytes in the message issued that is less than or equal to the allowed (defined) message for the last slot. It is not prohibited for other slots to take in aperiodic requests (especially slots that have larger cycles than Tcycle).
- The indirect action procedure on a slot can transmit the data of a slot that is not in the AC provided that the length of the data is not greater than the length of the data of the indirect slot (which, for reasons of determinism, cannot change the time period of a slot without a reconfiguration of the AC). The system may have a maximum number of slots available, but the AC may use fewer. The remaining slots can be used for indirection.

**Figure 4:**
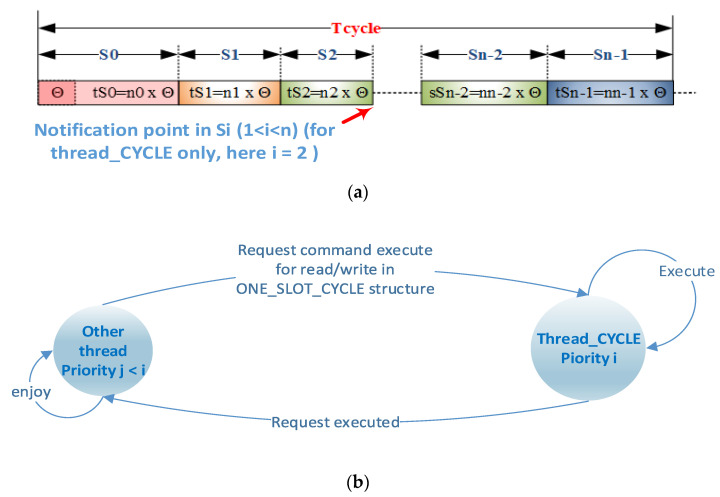
Slot notification point and a mutual exclusion method for ONE_SLOT_ENTRY. (**a**) Notification point at the end of the slot; (**b**) using flags, event, etc. to avoid race condition for access ONE_SLOT_ENTRY.

With the exception of Slots 0 and 1, which only have messages issued from the gateway, the other slots, except those indirect to Slot 1, have both a gateway message, hereinafter referred to as a request message, and a response from the server station (slave), hereinafter referred to as a reply message. The structure of these messages is presented in Figure 2. During the AC execution, only the thread dealing with the implementation of AC (*thread CYCLE*) can have write access to the members of the structure presented above. For this purpose, by means of, e.g., a flag, event, or signal mechanism specific to a specific RTOS, a thread may require *thread CYCLE* to execute at least the following commands (see Figure 4b):

- Disable/activate slot, which reads write values from the data area of the slot.
- Set the alternate slot, which can replace the current slot in the cycle with an alternate one outside the cycle (e.g., Location Index Slot 2 with Location Index Slot 5 or, e.g., *n* − 3, a position with Incremental Index 2, and the location index slot can later be replaced with the location index slot *m* + 3 < *n*, a position with Incremental Index 2).
- Notify the execution of a particular slot i, notify and activate a slot, and notify and activate the alternate slot, via which operations can be performed on the entry into the data structure of the slot, having a time Tcycle—*t_si_* (see Figure 4a). Notifications can be sent for or without running an alternative function on that slot once, a useful feature, for example, for setup from running.

**Figure 5:**
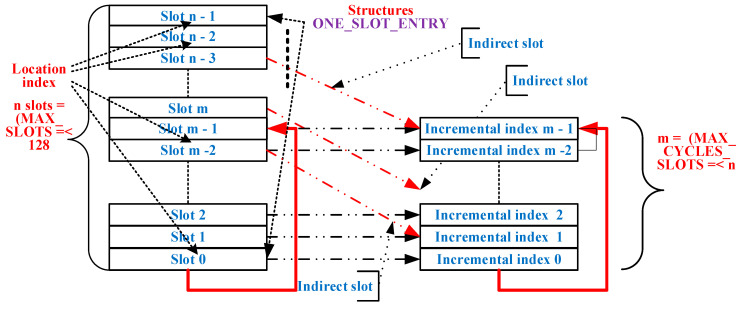
Slots and acquisition cycles.

Figure 5 shows all possible slots defined at the BSG level with indices of the location of the input into the structure area of that index and with the incremental index corresponding to the physical slot. In a physical slot, the location index (*AlternativeSlot*), which covers the range from 0 to MAX_SLOTS, does not have to coincide with the incremental index (*SlotAddress*) covering the range of consecutive values from 0 to MAX_CYCLE_SLOTS, for example, in the usual *AlternativeSlot* = *SlotAddress* structure. However, there may also be a situation where *AlternativeSlot* ≠ *SlotAddress*, in which case the *AlternativeSlot* value is taken into account if it is different from 0. As a result, an entry into the structure area that defines slots can be IN_CYCLE if its location index has a *SlotAddressi* pair (1 < *i* < m, Slots 0 and 1 of the AC have been excluded) and OUT_OF_CYCLE if it does not have such a pair.

### 3.3. Duration of an Acquisition Cycle

A ModbusE AC consists of *n* slots. As a result, the time period of a *t_CYA_* AC is as follows:

(6) $$t_{CYA}=\sum_{i=0}^{n-1}ts_{i}.$$

The period of *ts_i_* Slot consists of the following times:

(7) $$ts_{i}=tsw_{i}+thw_{i}+tcomm_{i}+tadjust_{i},$$

where *tsw_i_*, *thw_i_*, and *tcomm_i_* are defined as follows:

(8) $$tsw_{i}=tswC_{i}+tswS_{i},$$

(9) $$thw_{i}=2\times (thwC_{i}+thwS_{i}+tline_{i}),$$

(10) $$tcomm_{i}=tcommC_{i}+tcommS_{i}.$$

***tsw_i_ (tswC_i_, tswS_i_)*** The time required for software processing (to the client (master) and the server (slave)). Possible causes are the following: delay given by the interrupts handler (character or block level if DMA transfer is used); the time to switch two tasks if an RTOS is used (from the current task to the task that implements the ModbusE AC); time taken to calculate the CRC; the time it takes to transfer between buffers if applicable (from the reception buffer to the application buffer, or from the application buffer to the broadcast buffer); time required to prepare the slot for emission (indirect, checking, and updating the emission timers from k to k cycles, for creating history from j to j cycles, action on the status of a slot—activating/disabling the slot, activating/disabling indirection, activating/disabling read/write operation); time to update the result of the emission of a message (increment of the message counter issued on the slot, of emission errors); time to handle the response (incrementing message counter received by slot, errors); the time it takes to notify other event tasks (the slot has been activated/disabled, takes the value for the history); other implementation-specific processes.
***thw_i_ (thwC_i_, thwS_i_, tline_i_)*** Hardware delay times at master and slave, usually given by line drivers and other external intermediate logical gates if any, and internal specific to the µC used, plus the delay given by the communication line that depends on the physical distance relative to the device that emits (the master to which other devices to which the current device can subscribe) are added.
***tcomm_i_ (tcommC_i_, tcommS_i_)*** The times consumed by the characters issued by the master by the request message issued on each slot and those issued by the slave in response to these requests (except for Slots 0, 1, and indirect Slot 1).
***tadjust_i_*** For speeds of up to 115 kb/s, ModbusE you can also work with classic Modbus Slaves. For these there is no clear restriction on the response time to a master request. As a result, this time comes to help in adjusting the slot time period until the number of errors (signaled by the error counter at reception attached to that slot) meets a certain requirement (e.g., 1% errors/day or no error). There is no restriction on slave response time for ModbusE devices either because it is difficult to estimate it for the use of complex software. Again, tadjust_i_ comes to help solve this problem (device designers implementing the ModbusE protocol must take into account that it can operate up to speeds of 27 Mb/s and, as a result, a high response time of a station cancels out the advantage of this high communication speed).

For the time spent with the actual communication, the previously entered θ is used, which is the bit time period of a transmitted character. For ModbusE messages, the following general equations can be written (*nchC_i_* and *nchS_i_* represent the number of characters of the type given in the slot, i.e., without the slot address and CRC):

(11) $$tcommC_{i}=\left[htslot_{i}+3\times o+nchC_{i}+\left(1.5\times o\times \left(nchC_{i}\right)\right)+3.5\right)]\times \left(nbht+nbdata\right)\times \theta \;\;=\left[6.5+3\times o+nchM_{i}+1.5\times o\times nchM_{i}\right]\cdot \left(nbht+nbdata\right)\times \theta .$$

If *o* (optional = 0), the equation becomes (*htslot* = 3, 1 ch slot number, 2 ch CRC), and 3.5 ch the end of the message.

(12) $$tcommC_{i}=\left[6.5+nchM_{i}\right]\times \left(nbht+nbdata\right)\times \theta .$$

(13) $$\begin{array}{l} tcommS_{i}=\left[htslot_{i}+3\times o+nchS_{i}+\left(1.5\times o\times \left(nchS_{i}\right)\right)+3.5\right)]\times \left(nbht+nbdata\right)\times \theta =\\ \left[6.5+3\times o+nchS_{i}+\left(1.5\times o\times \left(nchS_{i}\right)\right)\right]\times \left(nbht+bdata\right)\times \theta . \end{array}$$

If *o* (optional = 0), the equation becomes

(14) $$tcommS_{i}=\left[6.5+nchS_{i}\right]\times \left(nbht+nbdata\right)\times \theta .$$

As a result, for *o* = 1, *tcomm_i_* is

(15) $$tcomm_{i}=\left[13+6\times o+nchM_{i}+nchS_{i}+1.5\times o\times \left(nchM_{i}+nchS_{i}\right)\right]\times \left(nbht+nbdata\right)\times \theta .$$

For *o* = 0,

(16) $$tcomm_{i}=\left[13+nchM_{i}+nchS_{i}\right]\times \left(nbht+nbdata\right)\times \theta ,$$

where the components are defined as follows:
***htslot***Header and trailer slot = 1 + 2. One byte is the slot address and 2 16-bit CRC bytes.**1.5**The distance between two characters is 1.5 characters.**3.5**The distance between two messages is 3.5 characters.***nchC_i_***Number of customer-issued data characters (master).***nchS_i_***Number of data characters issued by slave.***nbht***Number of bits in header + trailer for a character (usually a start bit and a stop bit).***nbdata***Number of bits per character (usually 8 but ModbusE also allows 16, 32, and 64 bits per character).***o***Optional o = 1 for classic Modbus specific working speeds and 0 for extended working speeds, up to 27 Mb/s specific ModbusE (requires DMA transfer, with any small delays being picked up by tadjust_i_).**θ**Time period of a bit depending on communication speed (e.g., for 1Mb/s, θ = 1 µs).

Replacing the times in Equations (6) and (7) with the values in the Equations (8)–(16), we get the following equation:

(17) $$t_{CYA}=\sum_{i=0}^{n-1}\{tswM_{i}+tswS_{i}+2\times (thwM_{i}+thwS_{i}+tline_{i})+\left[\left(13+6\times o+nchM_{i}+nchS_{i}+1.5\times o\times \;\left(nchM_{i}+nchS_{i}\right)\right)\times \left(nbht+nbdata\right)\times \theta \right]\}.$$

There is the possibility to write *t_si_* in the form

(18) $$ts_{i}=tcntrl_{i}+tpyld_{i},$$

where the components are defined as follows:
*tcntrl_i_*Time given by control operations and bits used by these operations.*tpyld_i_*Time load is the time needed to transmit payload data.

As a result, it is possible to rewrite Equation (17) if Slots 0 and 1 have no response.

(19) $$\begin{array}{ll} t_{CYA}=\sum_{i=2}^{i=n-1} & [tswM_{i}+tswS_{i}+2\times (thwM_{i}+thwS_{i}+tline_{i})\\ & +\left(13+6\times o+1.5\times o\times \left(nchM_{i}+nchS_{i}\right)\right)\times \left(nbht+nbdata\right)\times \theta \\ & +\left(nchM_{i}+nchS_{i}\right)\times nbht\times \theta +tadjust_{i}+\left(nchM_{i}+nchS_{i}\right)\times nbdata\times \theta ]+t_{S0}+t_{S1}. \end{array}$$

The last term in Equation (18) is *tpyld_i_*. If *o* is 0, the ModbusE case, the equation becomes

(20) $$t_{CYA}=\sum_{i=2}^{i=n-1}\left[tswM_{i}+tswS_{i}+2\times (thwM_{i}+thwS_{i}+tline_{i})+\left(13\right)\times \left(nbht+nbdata\right)\times \theta \;\;\;\;\;\;\;\;\;\;\;\;+\left(nchM_{i}+nchS_{i}\right)\times nbht\times \theta +tadjust_{i}+\left(nchM_{i}+nchS_{i}\right)\times nbdata\times \theta \right]+t_{S0}+t_{S1},$$

where *t_Sj_*, *j* = {0, 1}, can be written as

(21) $$t_{Sj}=tswC_{j}+thwC_{j}+tline_{j}+\left(6.5+3\times o+j+1.5\times o\times j\right)\times \left(nbht+nbdata\right)\times \theta +tadjust_{i}.$$

For *o* = 0, the equation becomes

(22) $$t_{Sj}=tswC_{j}+thwC_{j}+tline_{j}+\left(6.5+j\right)\times \left(nbht+nbdata\right)\times \theta +tadjust_{i}.$$

## 4. MBE Implementation Aspects

**Figure 6:**
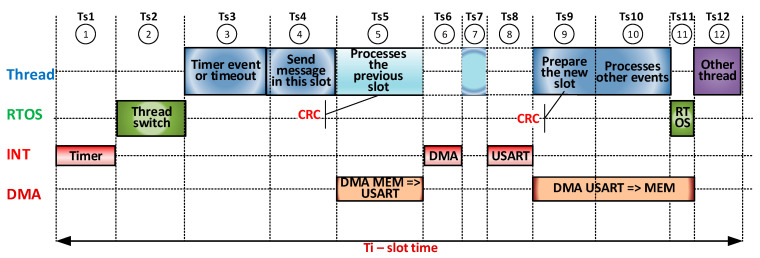
Pipeline SW (thread, RTOS, and ISR)/HW (DMA)—Scenario 1.

For specific Modbus RTU communication speeds from 9.6 kb/s to 115.2 kb/s corresponding to practical implementations, the implementation does not pose major designing issues. The interrupt service routine (ISR) can be used for the issuing of requests and the reception of responses (client gateway) or for receiving requests and issuing responses (server). A peculiarity for servers is that the server responses are broadcast and can be captured by all other servers (slave) connected on the same bus with two twisted wires. For speeds greater than 1 Mb/s (up to 27 Mb/s, the range of serial communication speeds explored in this paper), the duration of a serial communication bit may vary from 1 µs to 0.037 µs, which leads, for example, to two 13-frame MBE messages (1 slot address, 2 CRC, and 10 data) at an ideal transmission/reception time from 282 µs (1 Mb/s) to 10.59 µs (27 Mb/s), and the time distance character that generates a break for each frame (character) received/issued can be from 11 µs to 0.4 µs. These times can be stressful for an access gateway that can deploy, on the same µC and a Modbus TCP/IP or Modbus RTU server, multiple transactions and implement Modbus RTU functions under the control of an RTOS. For this reason, implementation should make intensive use of DMA transfer, interrupts, and possible Modbus facilities made available by the hardware architecture of the µC. CRC calculation or moving messages from the communication area to the processing area should not be forgotten if necessary. Scenarios are presented below for overlapping software (SW)/hardware (HW) operations in a kind of SW/HW pipeline. A first SW/HW pipeline implementation scenario is illustrated in Figure 6.

A few remarks on this scenario are the following:

- The interrupt generated by the timer (Ts1) signals the beginning of a new slot, and the event set by this interrupt activates the *thread CYCLE* run thread, released immediately by RTOS (Ts2) as the highest-priority thread. The ISR of this interrupt blocks all serial transfers from the previous slot.
- The *thread Cycle* deals with the timer event or a timeout event (Ts3), sends the request message attached to the slot using a nonblocking function that transfers via DMA with the generation of an interrupt at the end of the transmission (Ts4), processes the previous slot and calculates the CRC of the reply message received by it (Ts6, Ts7), prepares the new slot, executes commands from other threads (Ts9), and handles events from other threads and pass the thread waiting for the event from the timer (Ts10).
- It then transfers control of another thread by RTOS (Ts11) and runs the new thread Ts12.
- Interrupts given by DMA (TS6) and USART (Ts8) allow ISRs to manually change the communication direction (DMA activates the TC interrupt at USART), and the ISR for USART is designed to prepare the DMA and USART for the reception of the Ts8 response (if it is a long message, the calculation of the CRC can also be extended beyond the DMA response reception) Ti = sum *i* = 1–12 (*t_si_*).
- The proportions of the *t_si_* times in the figure are not representative. States 1 through 12 are a possible succession, but there may be other successions. If there are no events from the server status, State 10 disappears. If the message issued is high and the communication speed is quite low, the thread runs smoothly because State 5 (and by default State 8 and, in DMA only, States 9, 10, and 11) can be translated over time by States 10, 11, or 12.
- If the Ti slot is not set correctly, the timer interrupts can theoretically overlap the 2 states to 12. The operation will have slot-level errors.
- In State 5, which also involves calculating the CRC for the previous slot, if the message is long and the communication speed is high, the current slot period can be significantly extended.

**Figure 7:**
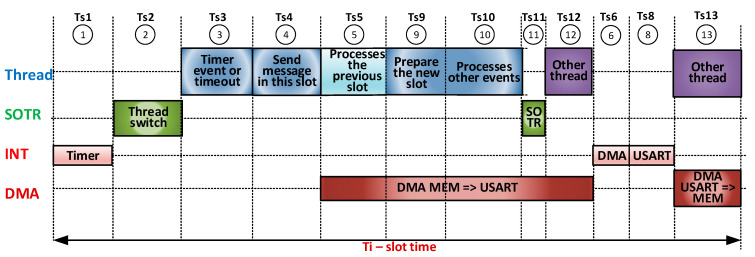
Pipeline SW (thread, RTOS, and ISR)/HW (DMA)—Scenario 2.

Figure 7 presents a second SW/HW pipeline implementation scenario. It is noted here that Ts6 and Ts7 have moved out of the execution of *thread CYCLE* because the current message is large. The time in the remaining free slot can be used by other threads.

Therefore, the following requirements for compatibility with the classic Modbus specification and ModbusE requirements must be taken into account for the implementation of the AC:

- Change the direction of the RS485 driver from reception to transmission and vice versa. This involves identifying the end of the broadcast message. The DMA-controlled emission provides an appropriate time period of 1.5 characters between two characters.
- In ModbusE, the presence of slots makes it possible to identify at the gateway a maximum duration of 1.5 characters between two consecutive characters of the reception message, as well as a duration of 3.5 characters, which signals the end of the message (with slot address, length, and CRC, one can easily verify the accuracy and presence of the received message, thus avoiding additional interrupts given by the DMA controller at the end of the message that can require significant additional time at high working speeds of more than 10 MB/s). This cannot be avoided at the level of the slave station, which must track all messages containing its own message(s), as well as the messages to which it has subscribed and Slot 0 or 1, e.g., the launch of the acquisition task.

As a result, the following factors are included in the implementation of the AC: *thread CYCLE* (set with the highest priority in the system), an RTOS that schedules the launch of the threads in execution, as well as the mechanisms of synchronization and communication between threads, the interrupts launched by the timer for the duration of a slot, DMA indicating that the timer attached to the emission message reaches 0, and USART for switching the direction of the RS485 driver back to reception and for launching the operation to receive the response message from the slave, if any.

### 4.1. Performance Evaluation of the Proposed MBE Solution

In this section, we aim to evaluate the performance implementation of the MBE concept proposed in Section 4, on the basis of the equations presented in Section 3.

We are interested in answering the following questions: What is the bandwidth of the serial communication channel? How much of this bandwidth is used to transport payload data? What is the usability of a slot time? What are the factors that have a great influence on the parameters previously mentioned? Proposals for optimizations and improvements are made on the basis of the results of the experiments.

### 4.2. Organizing the Experiment

For the implementation of the experiment, we used the following:

- Two MCBSTM32F400 development kits (Cortex-M4 µC at 168 MHz, 1 MB flash, 196 kB SRAM).
- A digital oscilloscope, max 500 MHz with four channels, type PICOSCOPE 6404D (Pico Technology).
- An *mbeGATE* instance of a BSG with the following main features:
  ○ Implements the *thread CYCLE* thread, which handles AC, and the help threads that dispatch requests from the Modbus TCP/IP and Modbus RTU server via USB.
  ○ Performs data acquisition and saves them on MicroSD.
  ○ Modbus TCP/IP and Modbus RTU servers (via USB) with local implementation of Modbus functions.
- A Telnet server (optional for tests).
- MDK-ARM Professional Development Environment.

The test AC had 30 slots in cycles from 0 to 29, with messages of lengths described in Table 1 and calculated with Equation (3).

Corresponding to the example in Table 1, for ModbusE, we have the following data.
Maximum number of bytes payload in cycle (considered indirect from Slot 29 to a message of a 122-byte transaction)=1 + 14 + 34 + 74 + 90 + 504 + 24 × 122 = 3645B (3644B without the byte in Slot 1)Minimum number of bytes payload in cycle=3645B − 1B − 122B = 3522B (if the byte in Slot 1 has no meaning in the cycle and if the asynchronous slot is redirected to Slot 1—there are no asynchronous messages)Maximum number of bits in a character’s payload=3644 × 8b = 29152bMaximum number of control bits at a character level for all characters in the slot=3644 × 3 + [3(S0) +4(S1) + 28 × 6 (S2-s29)] × 11b = 3644 × 3 + 175 × 11 = 10932b + 1925b = 12857b (1607,125B)Maximum number of bits per cycle=29152b + 12857b = 42009b/11b = 3819 frames

It follows from the table that, of the 42,009 b, only 29,152 b are useful, i.e., 69.39% of the communication times, and only 69.39% carry the load. A slot (without S0 and S1) has 66 bits of control regardless of the length of the messages. Each frame in the data range adds 3 b control. A longer message results in more bits of control, as well as more bits of data. An MBE’s maximum useful communication time slot is (252 × 8)/(66 + 252 × 11) = 2016b/2838b = 71.03%.

### 4.3. Results of the Experiment

**Figure 8:**
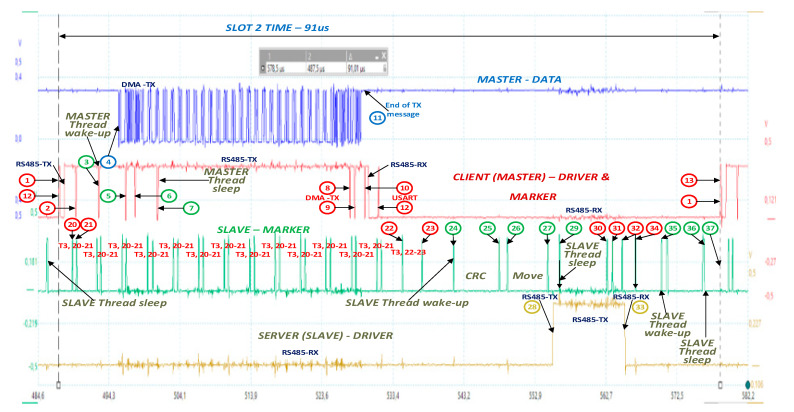
Measuring points, with an example for Slot 2, CLK 168 MHz, baud rate 10.5 Mbaud/s (slot time period: 91 µs).

Figure 8 shows the evolution over time during Slot 2. Points of interest obtained using a marker implemented on the switch signal of the direction of the RS485 driver are marked on the figure. Markers typically flag the entry and exit of an event (code sequences in *thread Cycle*, interrupts, and the activation/deactivation of the RS485 line driver). Start and stop bits mark the broadcast periods on the RS485 bus. The ISR marker for the timer that signals the start of a new slot is longer to make it easier to detect the start of a slot.

On the basis of the points indicated in Figure 8, measurable times were defined (Appendix A). Table 2 shows the measured values for Slot 3.

## 5. Discussions

The following is a comparison of message length using Modbus RTU, CAN, CAN FD, and Profibus DP-V0 protocols. As presented in Figure 2, the MBE message was introduced with support for ModbusRTU compatibility in the sense that the SYNC character also has slot address significance, and it can also indirectly be in a slot that does not have an IN_CYCLE index (see Figure 5). This reduced the number of bits considered in [3] from 11 (SyncSlot) + 33 (1 slot number + 2 CRC) + 11 × *n* to 33 + 11 × *n*.

Compared to the Profibus DP = V0 protocol, the SRD messages of the MBE protocol are shorter. The frames for 8b data are the same as 11b (1b START, 1b EVEN PARITY, 1b STOP, and 8b DATA), while the number of control characters (header + trailer), which accompany the SRD message, can be 6 or 9, i.e., greater than 3 (1 × slot address + 2 × for CRC), as required for MBE. Both protocols use a delimiter, 33b (three frames) and 3.5 frames, which makes it necessary to increase the control bits for MBE by 3.5 − 3 = 0.5 frames (5.5 b). Compared to the CAN protocol, calculation can be performed in bits as follows:

(23) $$47+⎡\frac{n}{8}⎤\;47+8n\;\left(\mathrm{CAN}\right)-33-11n\;\left(\mathrm{MBE}\right)=14+⎡\frac{n}{8}⎤47.$$

When *n* = 0–4, the difference is positive; when *n* = 5, the difference is negative. When *n* = 8, the difference is positive; when *n* = 15, the difference is positive again. When *n* = 16, the difference is positive; when *n* = 23, the difference is positive again. Therefore, CAN has the shorter message only when *n* = 5, 6, or 7; otherwise, the MBE message is shorter. Compared to the CAN FD protocol, we have

(24) 62(67 n>16))+padding+8n (CAN)−33−11n (MBE)=29 (34)+padding−3n.

The message length for CAN FD can range from 0 to 8, whereas, in the normal CAN protocol, the message length is 12, 16, 20, 24, 32, 48, or 64 (for fixed values, one can achieve a standard length (padding)). These values are transported unnecessarily, and padding bits are added to the control bits. In a message of not more than 64 bits, on the basis of the equations for the two protocols, it can easily be shown that, when *n* = 0–11, 13, 14, 17, 25, 26, 33–38, and 49. Of the 64 CAN values, FD is shorter than the 41 *n* values; thus, overall, it is shorter in 64% of cases. However, CAN FD is a protocol only for Levels 1 and 2 of the ISO-OSI model. If an implementation such as CANOpen FD is considered, the USDO protocol adds 14 bytes to each message, which leads to the modification of previous assessments reported to CAN FD in favor of MBE.

**Figure 9:**
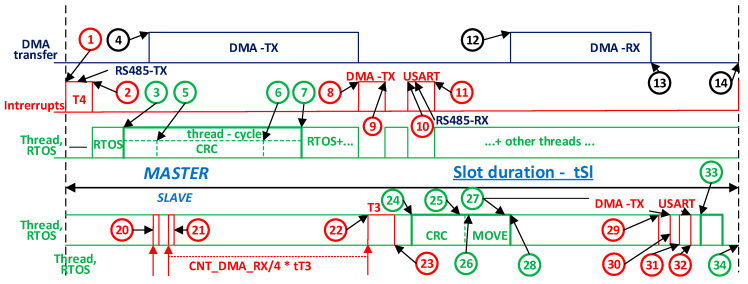
Time structure of a slot in the AC implemented with µC STM32F407 (168 MHz) for both client (MASTER) and server (SLAVE) (important and predictable periods).

In terms of maximum working speed, MBE has been tested at 27 Mb/s. CAN has rates of 1 Mb/s, Profibus has rates of 12 Mb/s, and CAN FD has rates of 8–10 Mb/s in the data cycle. From the point of view of access to the environment, CAN and CAN FD have an arbitrary mechanism of access to the multiple access environment with collision avoidance, while MBE and Profibus use TDMA (time division multiple access). In terms of the management of CAN and CAN FD, errors have evolved mechanisms, while Profibus and MBE have mechanisms provided by UART (universal asynchronous receiver). From an application point of view, MBE is an update for Modbus RTU. MBE allows the use/reuse of Modbus applications, which are used in low- (MBE/RTU) and medium-complexity (TCP/IP) applications at a low implementation cost, while maintaining the same ease of use, a low memory footprint, complexity scalability, domain and range, an ease of administration and improvement, and the same openness and inexpensiveness as those of MODBUS. The AC with the chosen cycle structure presented in Table 1, the measurement points indicated by the markers in Figure 8, the definitions in Appendix A, and the measured values in Table 2 is discussed below. According to the oscilloscope capture in Figure 8, the different levels of actions during execution for the chosen slot are shown in Figure 9. The total duration of the cycle period is 5666 ms, during which 3819 physical frames of 11b are transported on the physical medium for 70.62% of the cycle time, with this time period being managed by DMA channels without the intervention of the Cortex-M4 kernel, of which the load is 3644 B for 49.6% of the cycle duration. The time periods of Slots 0 and 1 (S0 and S1) are somewhat large compared to the small number of bytes transported on the physical environment, because the CRC of the aperiodic message (usually 134 B) is calculated during S0, and the indirectness is performed during S1, which in exceptional cases can have a response of up to 8 B.

The time period of the slot varies between 41.01 and 661 us depending on the size of the request/response messages. In this context, the time period of a slot can be used by the running threads of *thread Cycle*, which run on both the client and the server. Customer-level interrupts are generated by the timer that (1) measures the times of a slot (~2.8 µs), the DMA emission channel (~0.9 µs), and the USART programmed on the broadcast that signals the full transmission of the last character (the RS485 driver can be switched to reception) and (2) schedules its reception status (~1.3 µs—S0.1, and 2.1 µs elsewhere). This results in a total of 5.8 µs, i.e., 0.1% of the time period of the AC. Processing at the *thread Cycle* (tmhd) level varies between 2% (S6 having the longest time period consumed by DMA channels) and 46.76% (S0) of AC, with an average per AC of 9.2%, of which the most time-consuming routine is the one that calculates the CRC (between 0 (S1, S2) and 44.73 µs (S7)), with an average of 5.6% of AC. Switching threads due to *thread Cycle* lasts ~3 µs, with an average of 1.6% of AC.

A discussion of the MBE client (SLAVE) at the slot and AC level is also given. First, according to the analysis of the interrupt generated by the timer that generates the interrupt for the end of the message, it has a duration of ~0.8 µs if the received message is in progress and a duration of ~2.9 µs if a message end has been determined. Overall, the time spent by this operation is about 2% of AC. The message was also highlighted to move the message from the reception buffer to the buffer of the thread that processes the request, calculates the CRC, and sends a notification to the *thread Cycle* (server). Moving times are 0.8–41.25 µs, with an average of 5.6% of AC. Finally, the times (%mbusySlot) occupied by SW at the BSG (client) slots range from 2.52% (long messages) to 45.16% (short messages), with an average of 11.16% of the AC time period. At the server (SLAVE), these times are longer from moving the message to another buffer and range between 10.84% (long messages) and 30.60% (short messages), with an average of 14.31% of the AC level. Percentages allow time for other tasks to be scheduled for execution (in this experiment, there were 15 threads of execution in the system). This experiment was repeated using two 32F746GDISCOVERY Discovery kits with µC STM32F746, the difference being the communication speed of 27 Mb/s and the µC speed of 216 MHz. The AC time period decreased from 5.665 ms to 4.12 ms. Although the communication rate is 2.7 times higher, the working frequency of the kernel is 1.28 times higher, and there is extended support for MODBUS (most complete). The reduction of the AC time period is only 27%. The explanation lies in the adjustment time (*tadjust_i_*) that allows the other threads in the system to run. The total period of the cycle period is 4.12 ms, during which 3819 physical frames of 11b are transported on the physical medium for 37.8% of the cycle, this time period being managed by DMA channels without the intervention of the Cortex-M4 kernel, of which the load is 3644 B for 36% of the cycle.

Another experiment considered the idea of using a multicore µC—in this case, a combination of Cortex-M4 and Cortex-M0. Two Keil MCB4300 kits were used. The Cortex-M0 core was used only for MBE implementation, without RTOS, interrupts, or DMA. The chosen communication rate was 11.5 MHz at a frequency of 180 MHz of the CortexM0 core.

RTOS, DMA transfer, and interrupts were abandoned, and pooling was used instead. The AC time period decreased from 5665 ms to 4324 ms. The communication rate was 1.01 times higher, the working frequency of the nucleus was 1.07, and the reduction in the AC time period was 23.7%. The explanation lies in the absence of RTOS and ISRs. The total period of a cycle time is 4324 ms, during which 3819 physical frames of 11b are transported on the physical medium for 84.5% of the AC time period, with this time being managed by DMA channels without the intervention of the Cortex-M4 kernel, of which the load is 3644 B for 80.6% of the AC time. It follows from Table 3 that the two-core solution in which a kernel is dedicated to MBE has the greatest efficiency. It works in a loop and does not use RTOS, ISR, or DMA. In this case, the working frequency of the Cortex-M0 core copes with the transfer rate of 11.5 Mb/s, a character at 0.955 µs. The implementation of an HW/SW “pipeline” using DMA channels, by parallelizing the software operations needed to implement the AC with the serial communication controlled by DMA channels, has led to improved communication channel usage (37.8% for STM32F746). It has been shown that the efficiency of using a slot’s time decreases with increasing communication speed, but improves if the µC implementing MBE does not have other tasks. In other words, the adjustment time (tadjusti), invisible in the visualization of the temporal evolution within a slot, has a major influence and depends on the µC workload.

### 5.1. Description of Devices

FDI, FDT, and EDDL or the projects proposed in [25,26] may be solutions for describing networked devices that can be adapted for MBE. For sensors, the standard IEEE 1544.4, which is an emerging standard for adding plug-and-play capabilities to analog transducers, can be used. The basic mechanism for plug-and-play identification is the standardization of an electronic transducer data sheet (TEDS). A TEDS contains the critical information needed by a measuring instrument or system to correctly identify, characterize, interface, and use the signal from an analog sensor. TEDS is implemented for a sensor in one of two ways. First, TEDS can be stored in built-in memory, usually an EEPROM memory, in the analogue transducer as defined in IEEE 1451.4. Second, a virtual TEDS can exist as a separate, downloadable file from the internet. This virtual TEDS concept extends standardized TEDS benefits to old sensors and applications where built-in memory or EEPROM is not available. The IEEE 1451.4 defines the TEDS information encoding method for a wide range of sensor types and applications. To cover such a wide range while keeping the memory usage to a minimum, the IEEE 1451.4 TEDS uses the concept of templates that define the specific properties of different types of sensors. Another elegant concept is defined by the Foundation Fieldbus (FF) foundation, which uses function block, device description language, device description interoperability specification, transducer block common structure, pressure transducer block, communication profile, and more.

However, the simplest solution is the adaptation presented in [26] using the specifications of the CiA DS 306 series, with the stipulation that, for MBE, the section [COMMUNICATION] is not necessary.

### 5.2. Integrating BSG in IoT

The industrial Internet of things (IIoT) based on embedded systems or cyber physical systems (CPSs), running real-time applications for supervision, control, and monitoring of industrial processes, is designed to acquire data from and send data to sensors and transducers using LINWs. These data are sent to the application level where they can be distributed to the Internet using new Industrial IoT applications, SCADA applications [5], or smart building control applications. In this context, a fieldbus communication network has an important role in ensuring the support in order to transport these data.

The Modbus protocol with its RTU and TCP/IP variants is one of the oldest and most widely used, but it is only partially defined. For this reason, in this paper, an AC was implemented and evaluated for incomplete defined protocols using a proposed extension for Modbus, called ModbusE (Modbus Extension) [3], which is meant to achieve the Modbus RTU specifications and to increase its performance while maintaining compatibility with Modbus classic.

**Figure 10:**
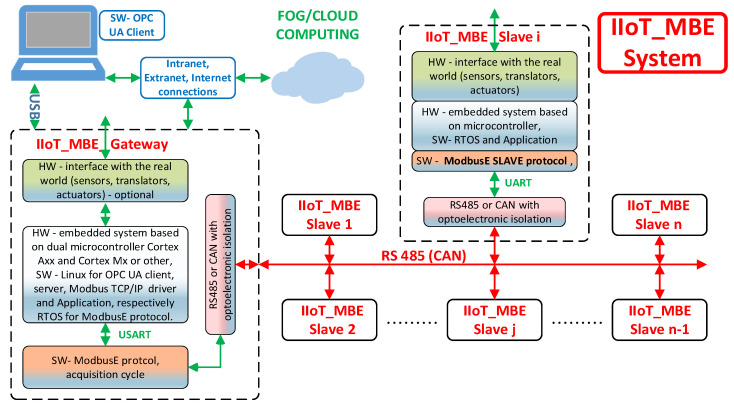
The main architecture of the experimental system for IIoT—ModbusE, (RTU) smart gateway.

In the future, improvements can be made to the protocol, e.g., defining a microMBE architecture for short and reliable links in which the slot address is 4 bits and the other 4 bits can define the length of the message, continuously or with predefined values (as in CAN FD), replacing the CRC with a simpler control amount (as in MODBUS ASCII or Profibus). The goal of this architecture is to design and develop a demonstrative experimental system for the implementation and testing of an intelligent gateway Industrial IoT: the Modbus Extension protocol for process monitoring and control management (IIoT MBE System: an application instance of BSG). This system will be used to evaluate and validate the AC and the extension ModbusE for the Modbus RTU protocol. The general architecture of the experimental demonstration system is presented in Figure 10.

The experimental demonstration IoT MBE System consists of a smart gateway IIoT MBE Gateway with three gateways (Modbus TCP/IP–ModbusE, Modbus RTU–ModbusE, and IIoT–ModbusE using the UPC UA server/client) and allows for implementing the acquisition cycle for ModbusE (AC_MBE) presented in this paper as part of the ModbusE protocol, the IIoT MBE Slave provided with the ModbusE server connected to the RS 485 communication network, the PC Modbus TCP/IP, and the RTU driver (Windows, Linux), which will include a call to a utility application for the ModbusE protocol (UAP_MBE) for configuration, validation, testing, and integration in Industrial IoT applications and a PC OPC UA client.

An interesting question that arises for high transfer speeds is that of who processes these data. The BSG-level TCP/IP server receives transfer requests from common external applications with access times of 500 ms. Parallelization can be done if multiple transactions are used (a maximum of 16 stipulated in the specification for Modbus TCP/IP). BSG may not have time for processing if it is based on a single µC. In some applications, the server stations (SLAVE) in the MBE can subscribe and use the information of any slot. BSG only provides CA management.

Another solution may be to use SoCs at the BSG level with Cortex-Ax, DSP, or Cortex-Mx, where Cortex-Mx (or similar) implements MBA CA, Cortex-Ax implements a calculation and control algorithm, and Cortex-Ax implements a server (possibly a client) OPC UA. Among many others, we mention the Texas Instruments Sitara AM5729, the Dual Arm Cortex-A15 microprocessor subsystem, 2 C66x floating-point VlIW DSPs, a 2× Dual Arm Cortex-M4 coprocessor, a 2× dual-core Programmable Real-Time Unit and Industrial Communication SubSystem (PRU-ICSS), used by BeagleBone AI, or STM32MP157 (where microprocessors are based on the flexible architecture of a Dual Arm Cortex-A7 core running up to 800 MHz, a Cortex-M4 at 209 MHz, and a CAN FD interface), used by DK32MP1157C.

## 6. Conclusions

This paper presented an original implementation of MBE in the form of BSG (MASTER) and server workstations (SLAVE). The MBE validation enabled the performance evaluation of this Modbus extension on a new message structure, an AC for obtaining a deterministic temporal behavior, a description of Modbus and MBE devices, and the definition of an architecture for integration into IIoT. Mathematical equations were defined, and specific times within an AC slot were calculated on their basis.

In the first two examples presented, the CPU workload was substantial, with 15 threads with stacks for USB and TCP/IP with applications (BSD sockets). In the example in which a microcontroller looped the implementation of MBE, the time efficiency of a slot was much better. FPGA implementation is expected to achieve the best results. To complete the MBE, a method of approaching the Modbus and MBE devices was also proposed. Another important observation is that the algorithm proposed in [3], on the slot time period, through network adjustments, is useful because the measurements taken in the experiment presented in the paper are not accessible for a simple network configuration process. In addition, few Modbus devices define a response time to Modbus requests. To this end, the theoretical communication time was decreased (69.39%), and a 100% adjustment time (empirical adjustment) was added, which was reduced or increased by a division algorithm with powers of 2, and a permitted error margin was taken into account. Since the experiment simulated all 30 slots on a single server card (SLAVE), this margin was 0. The criterion used was no error for 10 consecutive days (approximately 152,515,445 cycles). The algorithm in [3] was improved by a preassessment stage of server station response times. Different response times were simulated in the experiment. Random response times were also simulated, the conclusion being that, if these times have a sporadic occurrence, an acceptable margin of error can be allowed under the conditions of a specific application.

Lastly, it can be said that MBE is a simple extension that invigorates Modbus, retains compatibility, and can be used for low- and medium-complexity applications at a low cost. Because the MBE application level is based on the application level at MODBUS, MODBUS software is portable for Modbus stations and reusable with a simple wrapper for MBE.

## Figures and Tables

**Table 1:** The number of master and slave bytes for the 30 slots of the AC. Parentheses indicate control characters (2 × slot address + 2 × CRC16 (2 bytes) = 6 frames).

*i*	*nchC_i_*	*nchS_i_*	*sum*	*i*	*nchC_i_*	*nchS_i_*	*sum*	*i*	*nchC_i_*	*nchS_i_*	*sum*
0	0	No ACK	0(3)	1	0(1)	No ACK	0(4)	2	13	1	14(6)
3	29	5	34(6)	4	61	13	74(6)	5	61	29	90(6)
6	252	242	504(6)	7–28	61	61	122(6)	29	61 or Slot 1 indirect	61 No ACK	122(6) (4)

**Table 2:** Measured times for Master AC slots. (1) Implementation (µs), along with markers (cycle time period = 5,667,326 ms). The times were read from the oscilloscope software (STM32F407, CLK = 168 MHz, speed communication = 10.5 Mb/s).

Symbol/Slot	Slot 0, 3/0	Slot 1, 4/0	Slot 2, 16/4	Slot 3, 32/8	Slot 4, 64/16	Slot 5, 64/32	Slot 6, 255/255	Slot 7, 64/64	Slot 8–29, 64/64	%Cycle, 5665.97 µs
*t_si_*	41.01	42	64.01	90.95	144	161	661	194	194	
t_mintT4i_	2.496	2.457	2.482	2.461	2.415	2.464	2.513	2.479	2.458	~1.30%
t_mswitchi_	2.981	3.054	3.002	3.016	2.958	3.061	3.012	3.053	3.019	~1.60%
t_mthd1i_	3.522	-	-	3.851	3.831	3.85	3.837	3.866	3.844	~1.89%
t_mCRCi_ (-int. DMA-int. USART)	14.08 (−0.8937 −1.269) = 11.917	0	0	1.223	1.943	3.38	5.833	44.73	11.31	~5.60%
t_mthd2i_	3.432	-	-	3.227	3.291	3.219	3.282	3.272	3.201	~1.60%
t_mthd_ (-int. DMA-int. USART)	21.34 (−0.8937 −1.269) = 19.177	8.87−0.832−1.178 = 6.86	6.232	8.263	9.095	10.47	12.95	41.85	18.36	~9.20%
t_mintDMAtx_	0.8937	0.8319	0.8251	0.8249	0.818	0.8319	0.818	0.8111	0.859	~0.45%
t_mintUSART_	1.269	1.178	2.066	2.052	2.048	2.1	2.052	2.059	2.066	~1.06%
t_mtransi_	26.81	14.39	27.52	44.17	77.55	77.73	277.8	77.7	77.72	~41.18%
t_mreci_	14.2	27.6	36.52	46.78	66.41	83.29	383.2	116.3	116.3	~58.82%
t_mcommi_	2.857	4.409	17.02	33.75	67.3	67.28	267.4	67.28	67.29	~35.43%
t_swCi_	22.21	12.08	10.30	11.98	12.76	14.24	16.70	45.57	22.19	~11.16%
t_sint1T3_	0.839	0.873	0.671	0.774	0.817	0.782	0.771	0.761	790.3	~0.42%
t_sint2T3_	2.863	2.939	2.828	2.823	2.878	2.877	2.868	2.899	2.884	~1.53%
m_i_	6	6	10	13	23	23	81	23	23	691
t_sswitch1i_	4.028	3.82	4.039	3.617	4.201	3.83	4.136	4.213	3.646	~1.98%
t_sCRCi_	1.088	1.227	3.163	6.13	11.77	11.1	42.92	11.22	11.29	~1.89%
t_smovei_	0.780	0.908	2.805	5.447	10.55	10.53	41.25	10.56	10.54	~5.94%
t_sthdi_	13.23	9.421	9.22 + 5.948 = 15.168	14.78 + 5.991 = 20.771	25.41 + 5.942 = 31.352	24.94 + 5.962 = 30.902	87.45 + 5.909 = 93.359	25.05 + 6.003 = 31.053	24.88 + 5.976 = 30.856	~5.55%
t_sintDMAtx_	0	0	0.8762	0.9815	0.9151	0.939	0.9289	0.929	0.928	~0.45%
t_sintUSART_	0	0	2.001	2.048	2.059	2.041	1.996	2.066	2.052	~1.01%
t_sswitch2i_	0	0	3.843	3.374	4.048	3.844	3.815	3.383	3.84	~1.88%
t_stransi_	0	0	19.23	28.45	48.28	64.27	360.3	97.41	97.79	~48.88%
t_sreci_	13.23	9.421	6.721 + 38.03 = 44.751	8.146 + 54.33 = 62.476	7.168 + 88.68 = 95.848	11.15 + 85.61 = 96.76	12.19 + 288.4 = 300.59	8.596 + 87.97 = 96.566	10.91 + 85.33 = 96.24	~50.07%
t_scommi_	0	0	4.409	8.671	17.03	33.73	267.4	67.27	67.27	~33.15%
t_swSi_	12.55	8.82	15.46	20.27	27.74	27.29	71.63	27.48	27.25	~14.36%

**Table 3:** Summary of experiments.

HW Support	Communication Rate (Mb/s)	Communication Efficiency (3819B) %	Efficiency Payload (3644B) %	SW Implementation Type	Core/Multicore	Cost
MCBSTM32F400 (STM32F407) 168 MHz	10.5	70.62	49.6	RTOS, ISR, DMA	Cortex-M4	Small
32F746G DISCOVERY (STM32F746) 216 MHz	27	37.8	36	RTOS, ISR, DMA	Cortex-M7, support Modbus	Medium
MCB4300 (LPC4357) 180 MHz	11.5	84.5	80.6	Loop testing only (pooling)	Cortex-M4, Cortex-M0 (was used Cortex-M0)	Big

## Data Availability

Data sharing not applicable.

## Appendix A

This appendix contains the meaning of times according to the points defined in Figure 8.

**Table A1:** MBE slot times.

Symbol	Signification	Start/End Marker Points
t_si_	Slot *i* time period.	1–13
**For CLIENT (MASTER)—Slot i**		
T_ssl_	The moment the slot starts.	1
T_fsl_	The moment the slot ends.	13
t_SI_ = T_ssl_ − T_fsl_	Slot time period.	1–13
t_mintT4i_	Handler time period for interrupt generated by timer 4 that signals the start of a slot.	1–2
t_mswitchi_	Time period of switching to the *thread Cycle* task dealing with slots (the most priority in the system).	2–3
t_mthd1i_	Time 1—local *thread Cycle* (update of variables, launch of message transmission for the new slot—point 4, processing information related to the old slot—block 1 of code).	3–5
t_mCRCi_	Time to calculate the CRC for the message received in the previous slot.	5–6
t_mthd2i_	Time 2—local *thread Cycle* (treating information about the old slot—part 2, prepares the new slot for execution, takes an order sent through the event by the dispatcher thread). At the time 7, the thread *thread Cycle* switches on hold waiting for an event from the timer (4) that indicates the beginning of a new slot.	6–7
t_mthd_	Total time spent in a *thread Cycle* thread slot (sum of the last three previous times).	3–7
t_mintDMAtx_	Time period of handler (ISR) for DMA interrupt that announced the end of DMA transfer.	8–9
t_mintUSART_	The handler period (ISR) for the interrupt generated by USART that says it has finished transmitting the last character. All this handler prepares, if necessary, without Slots 0, 1, the reception of the reply. This is also where the RS485 driver is switched to reception.	10–12
t_mtransi_	Time period allocated to MASTER’s emission of the application.	1–12 or 1–7
t_mreci_	The time allotted to receiving the reply by MASTER if there is.	12–13 or 7–13 (short message issued)
t_mcommi_	Time period of broadcast of the message with DMA by MASTER to SLAVE.	4–11
%_mbusySlot_	Percentage of slot occupied by master AC management activities (RTOS, thread, and interrupts).	
**For SERVER (SLAVE—Slot i)**		
t_sint1T3_	The handler time period (ISR) for the interrupt generated by timer 3 for unsuccessfully testing the end of the message received by SLAVEi.	20–21
t_sint2T3_	Handler time period (ISR) for interrupt generated by timer 3 for successful end testing of the message received by SLAVEi.	22–23
m_i_	Number of failed tests for SLAVEi’s reception of the MASTER message.	
t_sswitch1i_	Time period of switching to the *mbSlaveThreadProtocol* task dealing with slots (the most priority in the system).	23–24
t_sCRCi_	The calculation time of the CRC for the message received by the slave for the current slot.	24–25
t_smovei_	How long the slave moves the message from the reception buffer to the final buffer.	26–27
t_sthdi_	How long *mbSlaveThreadProtocol* is active, reception, processing and emission (24–29), and reception preparation (35–36).	24–29 + 35–36
t_sintDMAtx_	Handler time period for discontinuation generated by DMA with the broadcast of the entire message from slave to master.	30–31
t_sintUSART_	The time period of the handler (ISR) for the interrupt generated by USART which says it has finished transmitting the last character. This is also where the RS485 driver is switched to reception.	32–34
t_sswitch2i_	Time period of switching to the *mbSlaveThreadProtocol* task dealing with slots (the most priority in the system).	34–35
t_stransi_	Time period allocated to the emission by SLAVE of the response if applicable.	24–35
t_sreci_	Time allotted to receipt of the request by SLAVE.	35–37 + 20–24
t_scommi_	Time period of transmission of the message via DMA by SLAVE to MASTER.	28–33
%_sbusySlot_	Percentage of slot occupied by slave AC management activities (RTOS, thread and interrupts).	
